# Prognostic Relevance of CCDC88C (Daple) Transcripts in the Peripheral Blood of Patients with Cutaneous Melanoma

**DOI:** 10.1038/s41598-018-36173-x

**Published:** 2018-12-21

**Authors:** Ying Dunkel, Anna L. Reid, Jason Ear, Nicolas Aznar, Michael Millward, Elin Gray, Robert Pearce, Melanie Ziman, Pradipta Ghosh

**Affiliations:** 1 Department of Medicine, University of California, San Diego, La Jolla, California, USA; 2 School of Medical Sciences, Edith Cowan University, Perth, WA Australia; 3 Centre de Recherche enCancérologie de Lyon (CRCL), Lyon, France; 4 School of Medicine, University of Western Australia, Crawley, Australia; 5 Department of Medical Oncology, Sir Charles Gairdner Hospital, Nedlands, Australia; 6 School of Biomedical Science, University of Western Australia, Crawley, Australia; 7 Department of Cellular and Molecular Medicine, University of California, San Diego, La Jolla, California, USA; 8 Rebecca and John Moores Cancer Center, University of California, San Diego, La Jolla, California, USA

## Abstract

A loss of balance between G protein activation and deactivation has been implicated in the initiation of melanomas, and non-canonical Wnt signaling via the Wnt5A/Frizzled (FZD) pathway has been shown to be critical for the switch to an invasive phenotype. Daple [CCDC88C], a cytosolic guanine nucleotide exchange modulator (GEM) which enhances non-canonical Wnt5A/FZD signaling via activation of trimeric G protein, Gαi, has been shown to serve opposing roles–as an inducer of EMT and invasiveness and a potent tumor suppressor–via two isoforms, V1 (full-length) and V2 (short spliced isoform), respectively. Here we report that the relative abundance of these isoforms in the peripheral circulation, presumably largely from circulating tumor cells (CTCs), is a prognostic marker of cutaneous melanomas. Expression of V1 is increased in both the early and late clinical stages (*p* < 0.001, *p* = 0.002, respectively); V2 is decreased exclusively in the late clinical stage (*p* = 0.003). The two isoforms have opposing prognostic effects: high expression of V2 increases relapse-free survival (RFS; *p* = 0.014), whereas high expression of V1 tends to decrease RFS (*p* = 0.051). Furthermore, these effects are additive, in that melanoma patients with a low V2-high V1 signature carry the highest risk of metastatic disease. We conclude that detection of Daple transcripts in the peripheral blood (i.e., liquid biopsies) of patients with melanoma may serve as a prognostic marker and an effective strategy for non-invasive long-term follow-up of patients with melanoma.

## Introduction

Melanoma is a highly malignant cancer and causes more deaths than other forms of skin cancer; as in most cancers, early detection and surgical intervention are imperative for a curative outcome. However, in the case of melanoma, even with ‘curative’ surgical resection, the risk of recurrence is never completely absentand late recurrences, even beyond 8 years (y), can occur at a frequency ranging from 0.41% to 25% across different studies, with 6.8% at 15 y and 11.3% at 25 y^1,2^. The late recurrence population is particularly interesting to study as it represents a group of patients exhibiting tumor dormancy, defined as a stage in cancer progression in which residual disease is present but remains asymptomatic^2^. Hence, early detection of patients in this clinical stage of melanoma is of paramount importance so that they can be offered adjuvant therapy.

Tumor dormancy is best studied by examining patients who demonstrate persistent circulating or disseminated tumor cells (CTC or DTC) after removal of all clinically evident disease^3^. In fact, metastatic melanoma was the first malignancy in which CTCs were detected^4^. More recently, the presence of circulating melanoma cells (CMCs) in the peripheral blood of patients with metastatic cutaneous melanoma has been identified by detecting mRNA transcripts of specific melanoma markers. These specific transcripts from one or more CMCs in 10 ml of peripheral blood can be identified in 45% of all melanoma patients at varying disease stages (AJCC Stages I/II, III, and IV: 35%, 44%, and 86%, respectively)^5–9^. While the prognostic impact of the number of CMCs remains to be determined, the prognostic impact of detecting molecular markers from these CMCs in the peripheral blood of melanoma patients has been evaluated by multiple groups within the last decade. These studies are in general agreement that the detection of unique multi-panel biomarkers such as S100B, MAGE-3, p97, MUC-18, S100A4, MART1, HMB45, and tyrosinase, by real-time quantitative PCR (qRT-PCR) in CMCs of melanoma patients can have a valuable prognostic utility, especially in patients with Stage II and III disease^10–14^. These studies demonstrate that molecular detection of CMCs may be valuable to detect early disease recurrence and to stratify patients for adjuvant therapy. However, given the heterogeneous nature of melanoma cells, no single marker or panel of markers appears to be sufficiently robust in predicting outcome.

Despite these insights, the identification of CMC-biomarkers with strong rationale and mechanistic insights and within specific signaling pathways known to fuel melanoma metastasis,remains unexplored and unrealized. In this regard, several studies agree that activation of non-canonical Wnt signaling and inactivation of the canonical Wnt pathway is associated with metastatic features of melanoma and thus poor survival^15–17^. Here we report the identification of Daple (CCDC88C), a cytoplasmic transducer of Wnt signals^18–20^, as a candidate marker for monitoring deregulated Wnt signaling during melanoma progression. Daple links ligand-activated Frizzled receptors to Gi protein activation^18^. Such activation, on one hand antagonizes the canonical Wnt pathway and suppresses a neoplastic transformation and tumor growth. On the other hand, it enhances non-canonical Wnt pathway and Akt signals, triggering epithelial-mesenchymal transition (EMT) and tumor invasion^18^. Subsequent work unraveled that Daple-dependent modulation of Wnt signaling is triggered not just by Wnt ligands^18^, but also by growth factors (EGF)^20^ and junctions-associated Cadherin-catenin complexes that trigger the PI3K-Akt pathway^19^. Perhaps as a consequence of such convergent signaling, ourselves and others have demonstrated Daple’s role in fueling the metastatic progression of gastric^21^ and colorectal cancers (CRCs)^18,22^ and its ability to serve as a prognostic marker in the CTCs of patients with metastatic CRCs^22,23^. We hypothesized that detection of Daple transcripts in the peripheral blood of patients with melanoma may serve as a surrogate marker for deregulated Wnt signaling during melanoma progression. Our findings present evidence that tracking levels of its transcripts could serve as an effective strategy for non-invasive long-term follow-up of patients with melanoma.

## Results and Discussion

### Rationale for choosing Daple as a biomarker for metastatic progression of melanoma

**Figure 1:**
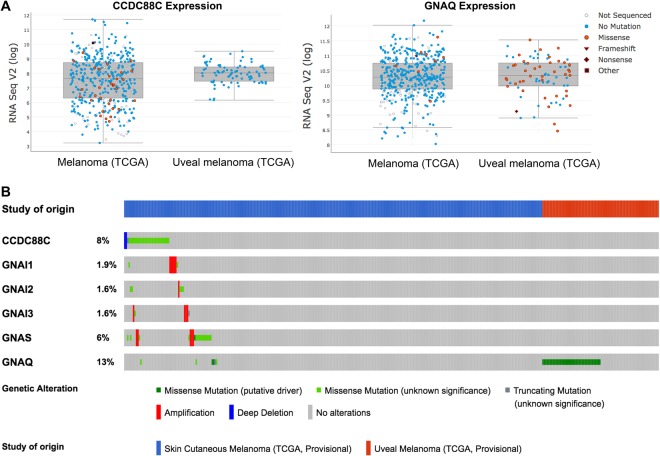
Daple expression is altered (deleted or mutated) in cutaneous, but not in uveal melanomas. (**A**) Graphs display the levels of expression of CCDC88C/Daple (*left*) and GNAQ (*right*) in cutaneous melanoma vs. uveal melanoma patients in The Cancer Genome Atlas (TCGA) dataset. Individual point represents mutational status of each patient. While CCDC88C mutations are restricted to cutaneous melanomas, GNAQ mutations are found predominantly in uveal melanomas. (**B**) Frequency of gene alterations in cutaneous melanoma vs. uveal melanoma patients in The Cancer Genome Atlas (TCGA) dataset is shown for CCDC88C (Daple) and heterotrimeric G-proteins (GNAI1, GNAI2, GNAI3, GNAS, and GNAQ). Mutations in CCDC88C and GNAI and GNAS proteins were observed exclusively in cutaneous melanomas. Mutations in GNAQ was predominantly observed in uveal melanomas.

We previously reported that the Disheveled-binding protein, Daple, is a guanine nucleotide exchange modulator (GEM) for heterotrimeric G proteins within the Wnt signaling cascade^18^. Subsequent work revealed that the human Daple/CCDC88C gene is expressed as two functional isoforms: Daple-full length (V1) (2028aa) and Daple-V2 (552aa)^22^. These isoforms serve contrasting roles during cancer progression; while both isoforms cooperatively antagonize Wnt signaling and suppress tumor cell proliferation and growth via their G protein binding and activation (GBA) motif^18^, only the full-length V1 isoform triggers EMT and invasion. In the colon, both isoforms collaboratively suppress tumor cell growth, and have an additive prognostic impact (worse outcome when both are suppressed). However, only Daple-V1 is increased in invasive tumor margins and in CTCs disseminated during CRC progression. We next chose to study the role of Daple in melanoma, which, like colorectal cancers, is another cancer that is characterized by dysregulation of heterotrimeric G-protein as well as Wnt signaling; prior studies have implicated both pathways in the metastatic progression of melanomas^24,25^. When we analyzed the RNA sequencing dataset from The Cancer Genome Atlas (TCGA) for dysregulation of Daple/CCDC88C expression (CNVs, deletions or mutations) and the frequently mutated heterotrimeric G-protein GNAQ^26^, we found that mutations have been identified in patients with cutaneous, but not uveal melanomas for Daple/CCDC88C. For GNAQ, on the other hand, although mutations have been identified in both cutaneous and uveal melanomas, these are predominantly found in uveal melanomas (Fig. 1A,B). Mutations found in Daple/CCDC88C included both missense and truncating mutations (Supplemental Figs S1 and S2). Notably, mutations in other heterotrimeric G-protein α-subunits (GNAI1, GNAI2, GNAI3, and GNAS) that are modulated by this family of GEMs (of which Daple is a member and CCDC88A/Girdin and NUCB1/2 are others^27^) were only found in cutaneous melanomas (Fig. 1B). These findings, along with the observation that Daple/CCDC88C was indeed abundantly expressed in distant metastases to diverse organs or sites (Supplemental Fig. S3), led us to hypothesize that dysregulated signaling via Daple/CCDC88C in cutaneous melanomas may have a role in the progression to metastatic disease. If so, that would imply that deregulated G protein signaling may be a common theme in both cutaneous and uveal melanomas–In uveal melanomas such deregulation is *directly* mediated by oncogenic mutations in the G protein α-subunit (GNAQ), whereas in cutaneous melanomas such deregulation may be *indirectly* mediated via mutations and altered expression of modulators of G proteins such as Daple-GEM.

### Transcripts of Daple-V1 (V1), but not Daple-V2, is increased in the peripheral circulation of patients with melanoma

**Figure 2:**
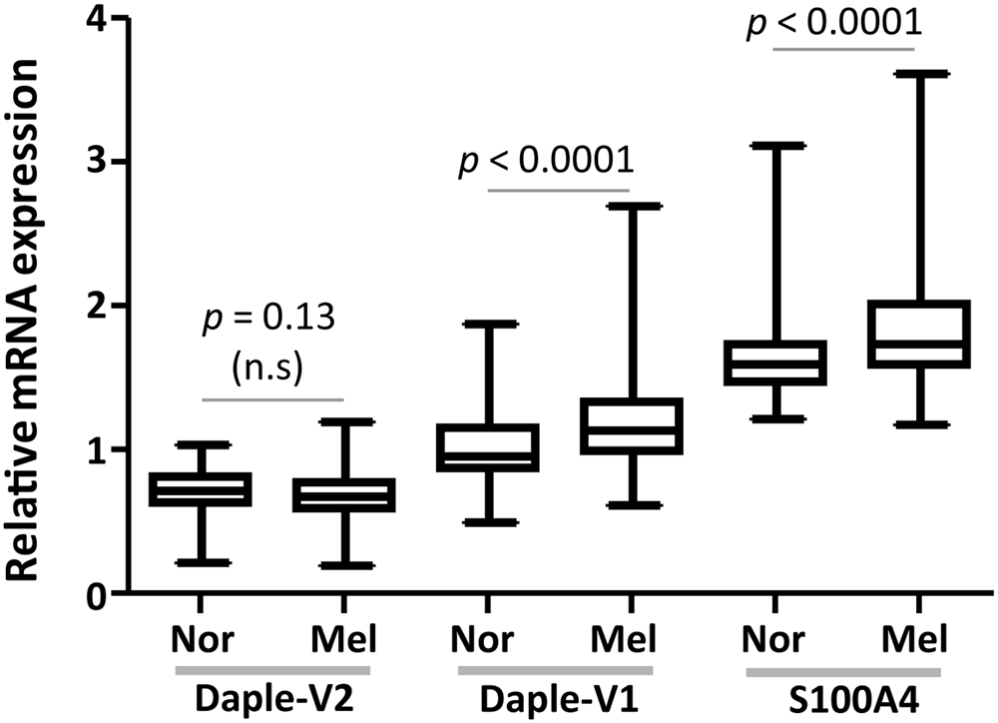
Comparison of the expression of Daple (isoforms V1 and V2) and S100A4 in the peripheral blood of patients with cutaneous melanoma *vs*. normal healthy subjects. Expression of Daple V1, V2 and S100A4 (positive control) mRNA were analyzed by qPCR in patients with melanoma (n = 205) and in normal healthy controls (n = 142). Findings are displayed as box plots for the relative mRNA expression of each gene normalized to GAPDH using standard curve method. The statistical significance of the difference for each gene between melanoma group and normal group was calculated by t-test. Levels of expression were significantly elevated for Daple-V1 and S100A4 (positive control), but not Daple-V2 in patients with melanoma compared to healthy normal subjects.

We recently showed that the expression of Daple full-length (Daple-V1) was elevated in EpCAM-immuno-isolated CTCs of colorectal cancer patients, and that Daple promotes EMT during tumor cell dissemination and disease progression^18,22^. Expression of Daple-V2, on the other hand, was found to be decreased in primary CRC tumors^22^. Because Daple-V1 and -V2 are differentially expressed in CTCs of CRCs patients, we first asked if this is also the case in patients with melanoma (see Table 1 for patient cohort characteristics). Because detection of gene transcripts in the peripheral blood is a sensitive surrogate marker of melanoma CTCs^8,28^, we analyzed the abundance of Daple transcripts in the peripheral blood of patients with melanoma (n = 205)and of healthy controls (n = 142) by qRT-PCR. We found that patients with melanoma have significantly elevated level of Daple-V1 expression compared to normal healthy controls (*p* < 0.0001; Fig. 2). The same pattern of expression was observed as for the melanocyte marker and the metastasis-associated protein, S100A4^29^, which is a transcriptional target of the Wnt/β-Catenin pathway^30^ and currently used as a marker to diagnose melanoma^31^. By contrast, no difference in Daple-V2 expression was observed between transcripts in peripheral blood of melanoma patients and the healthy controls (Fig. 2). These findings indicate that transcripts of full length Daple-V1, but not V2 are selectively upregulated in patients with melanoma and suggest that high levels of Daple-V1 may contribute to melanoma progression.

**Table 1:** Characteristics of patient cohort analyzed in this study.

Prognostic factor		Number of patients
Sex	Male	128
	Female	77
AJCC stage	0	37
	I	71
	II	37
	III	25
	IV	35
Primary site	Face	35
	Neck	4
	Torso	48
	Arms	43
	Legs	35
	Scalp	12
	Unknown	28
Breslow thickness (mm)	<1 mm	92
	1–4 mm	66
	>4 mm	24
	Unknown	23
Clark level	I	40
	II	26
	III	26
	IV	76
	V	12
	Unknown	25
Ulceration	No	163
	Yes	26
	Unknown	16

### Transcripts of Daple-V1 increase, but Daple-V2 decrease in the peripheral blood of patients during melanoma progression

**Figure 3:**
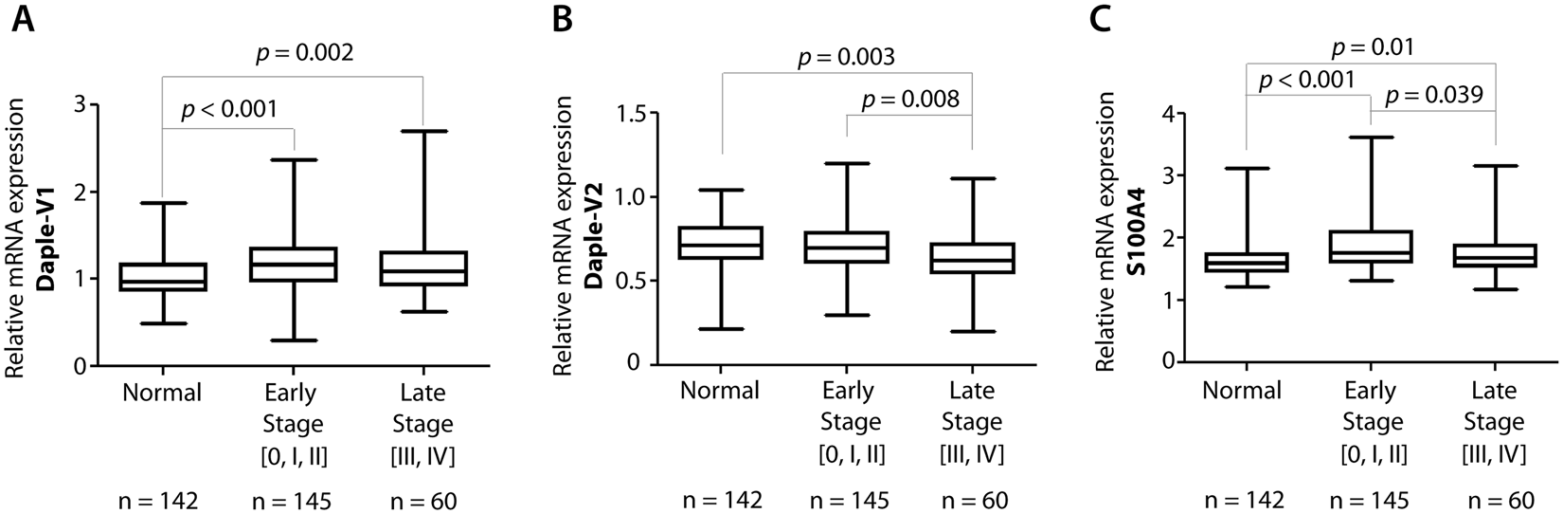
The expression of Daple isoforms V1 (**A**) and V2 (**B**) and S100A4 (**C**) in the peripheral blood of normal subjects and of patients with early or late-stage melanomas. Expression of Daple V1, V2 and S100A4 (positive control) mRNA were analyzed in patients with early (clinical stage 0, I, and II; n = 145) or late (clinical stage III and IV; n = 60) stage melanoma and in normal healthy controls (n = 142) by qPCR. Findings are displayed as box plots for the relative mRNA expression of each gene normalized to GAPDH as in Fig. 2. The statistical significance of the difference for each gene between groups with either early or late-stage melanoma and normal healthy controls was calculated by t-test. While transcripts of Daple-V1 (**A**) and S100A4 (**C**) are elevated in both early and late stage melanomas, those of Daple-V2 are reduced exclusively in late stages of melanoma.

**Figure 4:**
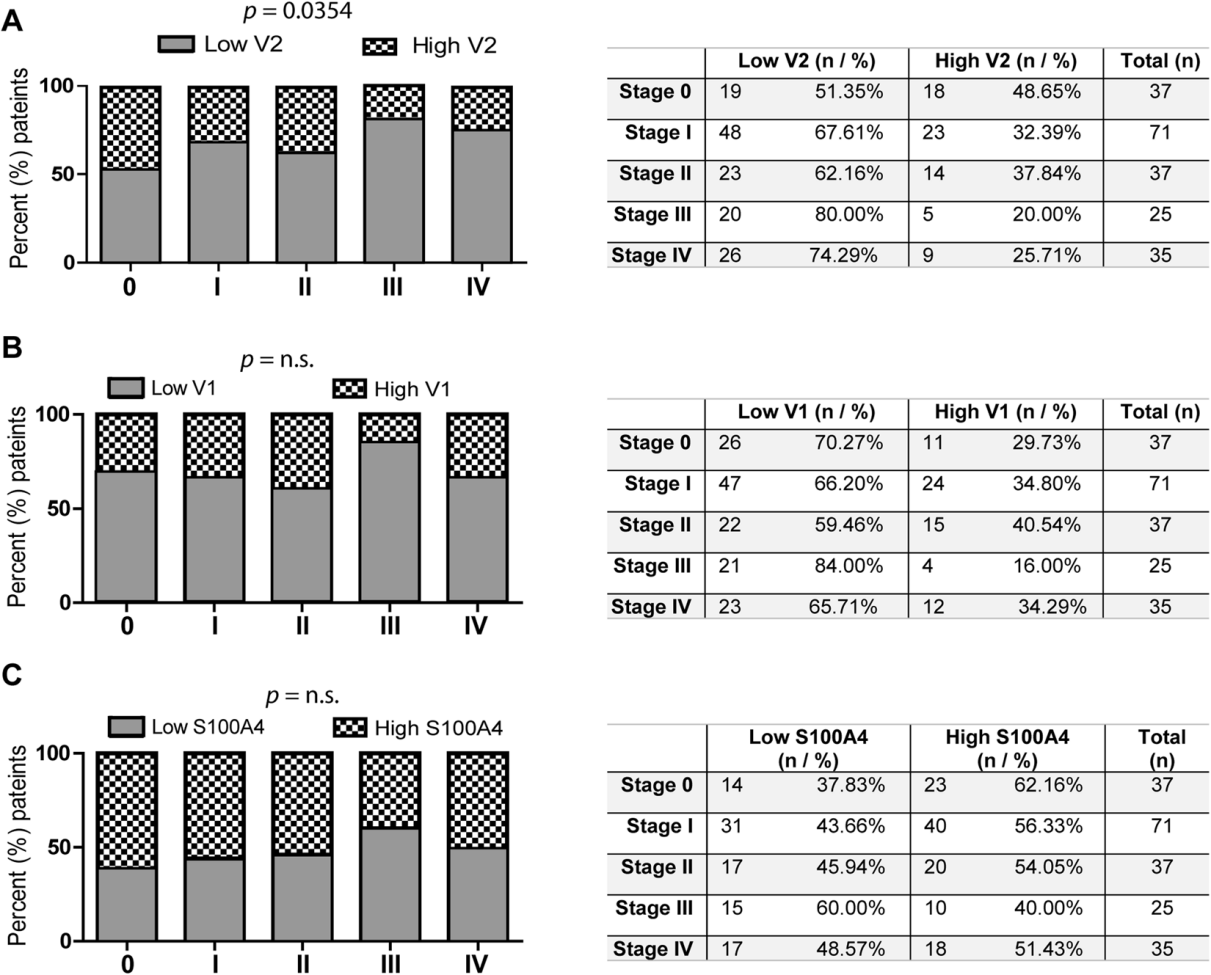
The association between the clinical stage of melanoma and the levels of expression of Daple isoforms V1 (**A**) and V2 (**B**) and S100A4 (**C**) in the peripheral blood. Expression level of Daple V1, V2 and S100A4 (positive control) mRNA were analyzed in patients with different stage (clinical stage 0, I, II, III, IV) melanoma by qPCR. Findings are displayed as bar graphs showing the proportion of patients (Y axis) in each stage with low *vs*. high expression of indicated genes. The statistical significance of the associations between stages and the expression levels of Daple-V1, V2 and S100A4 were calculated by Chi-square test for trend. The optimal cut-off values for high vs low gene expression levels were derived by maximally selected log-rank statistics performed using Cutoff Finder (R Software v2.15.0) (see Table 4).

To further dissect the patterns of expression of Daple-V1 and Daple-V2 relative to disease stage, we analyzed both isoforms in the peripheral circulation of patients with early stage (AJCC stages 0, I, II) vs. late stage (AJCC stages III, IV) melanomas. We found that Daple-V1 expression is significantly increased in both early (*p* < 0.001; Fig. 3A) and late (*p* = 0.002; Fig. 3A) stages compared to healthy controls. Expression of Daple-V2 is significantly lower in late stage melanoma compared to early stage disease and healthy controls (*p* = 0.008 and 0.003, respectively; Fig. 3B); no significant differences were noted between healthy and early stage melanoma patients. Levels of S100A4 expression, the positive control we used in this study, were increased in both early (0, I, II) and late (III, IV) stages (Fig. 3C) compared to normal healthy controls (*p* < 0.001 and 0.01, respectively). The changes in Daple expression we observe relative to disease stage are consistent with the patterns of Daple-V1 vs. V2 expressionin CRC stages; in both scenarios Daple-V1 increases but Daple-V2 decreases as the disease progresses from the early state to the late invasive stage and undergoes hematogenous dissemination. The change in these transcripts in peripheral blood may reflect the entrance of CTCs into the peripheral circulation^22^. We separated the patients with high expression of Daple-V1/V2 and low expression group in each clinical stage (stages 0, I, II, III, IV) based on the quantitative mRNA estimation by qPCR and looked for an association between clinical stage of melanoma and the level of expression of Daple-V1/V2. We found that only Daple-V2 levels significantly change with increasing clinical stage (p = 0.0354; Fig. 4A), which is consistent with the correlation analysis shown in Supplemental Table 3. This finding further suggests that Daple-V2 may play an important role during melanoma progression.

### Elevated Daple-V1 and suppressed Daple-V2 transcripts in the peripheral circulation of patients with early stage melanoma carries an increased risk for distant metastasis

**Figure 5:**
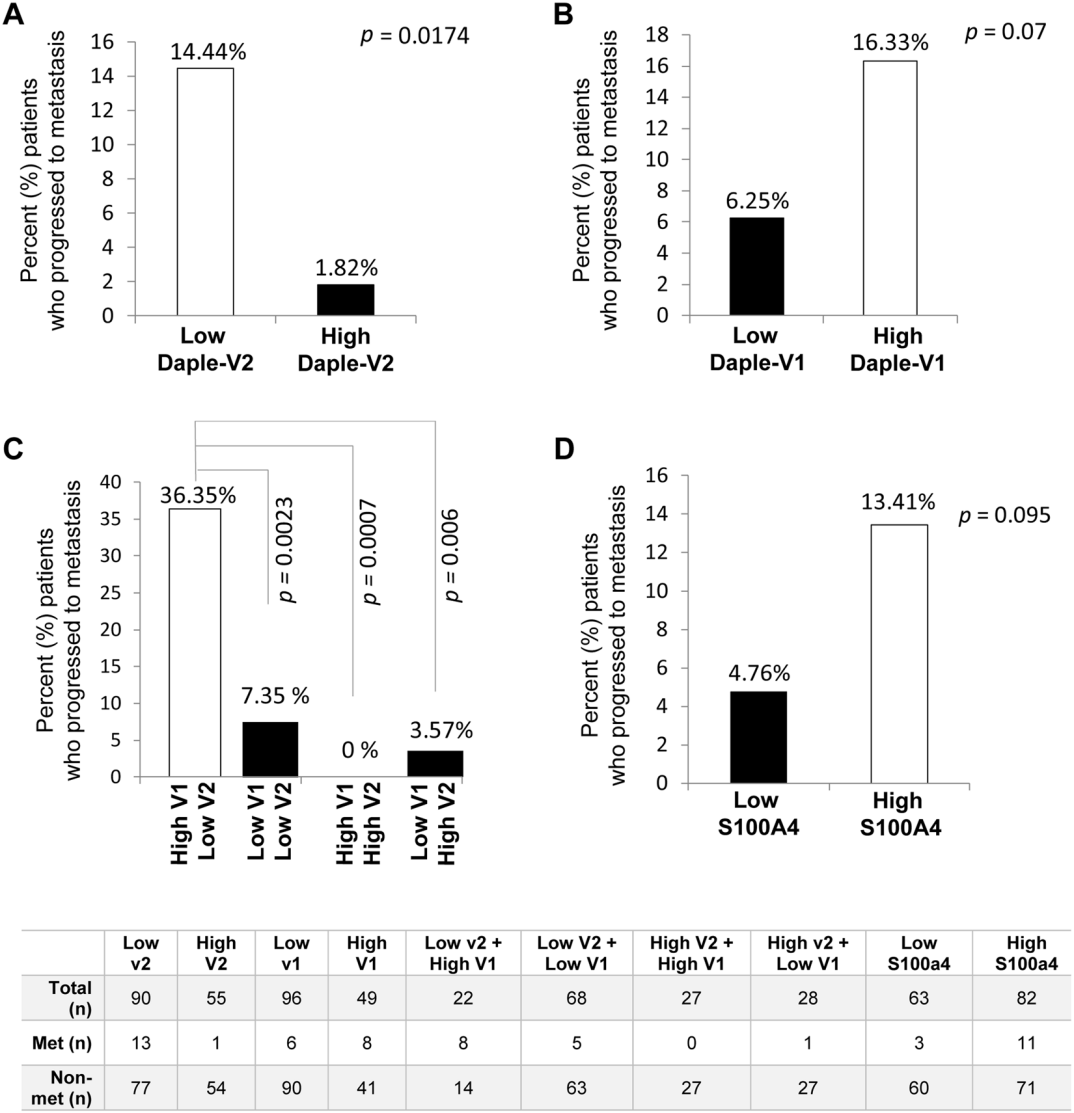
Frequency of progression to metastasis among patients stratified based on high *vs*. low levels of circulating transcripts of Daple-V2 alone (**A**), Daple-V1 alone (**B**), Daple V1 and V2 combined (**C**), and S100A4 (**D**) in the peripheral blood. Bar graphs display the proportion of patients who progressed to metastasis (Y axis) in patients with low *vs*. high expression of indicated genes. Fisher exact test was used to compare each subgroup. (**A**) Compared to those with high Daple-V2 expression (n = 55), those with low Daple-V2 (n = 90) expression displayed higher frequency of metastatic progression. (**B**) Compared to those with low Daple-V1 expression (n = 96), those with high Daple-V1 (n = 49) expression displayed higher frequency of metastatic progression. (**C**) Compared to those with low-V1-low-V2 (n = 68), high-V1-high-V2 (n = 27) and low-V1-high-V2 (n = 28) signatures, those with high-V1-low-V2 (n = 22) expression displayed higher frequency of metastatic progression. (**D**) Compared to those with low S100A4 expression (n = 63), those with high S100A4 (n = 82) expression displayed higher frequency of metastatic progression.

Next, we asked if the levels of expression of Daple-V1 and Daple-V2 in the peripheral blood of early stage melanoma patients can correlate with the frequency of progression tometastatic disease. In 145 patients with early stage melanoma, who progressed to metastatic disease later, we found that low expression of Daple-V2 was associated with a higher metastatic rate (14.44%) compared to high expression of Daple-V2 (1.82%; *p* = 0.0174; Fig. 5A). This pattern was not seen in the case of Daple-V1; in fact, high expression of Daple-V1 tracked with higher metastatic rate (16.33%) compared to low expression of Daple-V1, although that trend did not reach significance (6.25%; *p* = 0.073; Fig. 5B). When we compared the rates of metastatic progression among various groups with high vs. low Daple-V1 and/or V2, we found that the group with the signature of low Daple-V2 and high Daple-V1 progresses to metastasis more frequently (36.36%) compared to all other groups (Fig. 5C). These findings suggest that an analysis that accounts for the levels of expression of both Daple isoforms could perform better in prognosticating outcome than each one alone, i.e., Daple-V1 and Daple-V2 may have anadditive prognostic impact. The high expression of the metastasis-associated protein, S100A4^29^ was also somewhat indicative of a higher metastatic rate among the high expressing group (13.41%) compared to the low expressing group (4.76%; *p* = 0.095; Fig. 5D) but did not reach significance. We speculate that the prometastatic trends we observed in the case of both Daple-V1 and S100A4 did not reach significance because of insufficient patients in the cohort. Regardless, these findings are consistent with our recent findings that Daple-V2 is a potent tumor suppressor, whereas Daple-V1 triggers EMT and invasion^18,22^.

Finally, we also found that patients with low Daple-V2 transcripts in their peripheral circulation had higher levels of other melanoma cell markers (Fig. S4A,B; Supplementary Table S4; data from^7^), indicating that these patients may have higher numbers of circulating melanoma cells. Compared to patients with high Daple-V2, those with low Daple-V2 had a higher incidence of detectable ABCB5 (Fig. S4B), a melanoma stem cell marker; no relationships were observed with Daple-V1. Taken together, these findings suggest that Daple-V2 alone may serve as an effective prognostic marker in early stage melanoma.

### Prognostic impact of Daple-V1 and Daple-V2 in patients with melanoma

**Figure 6:**
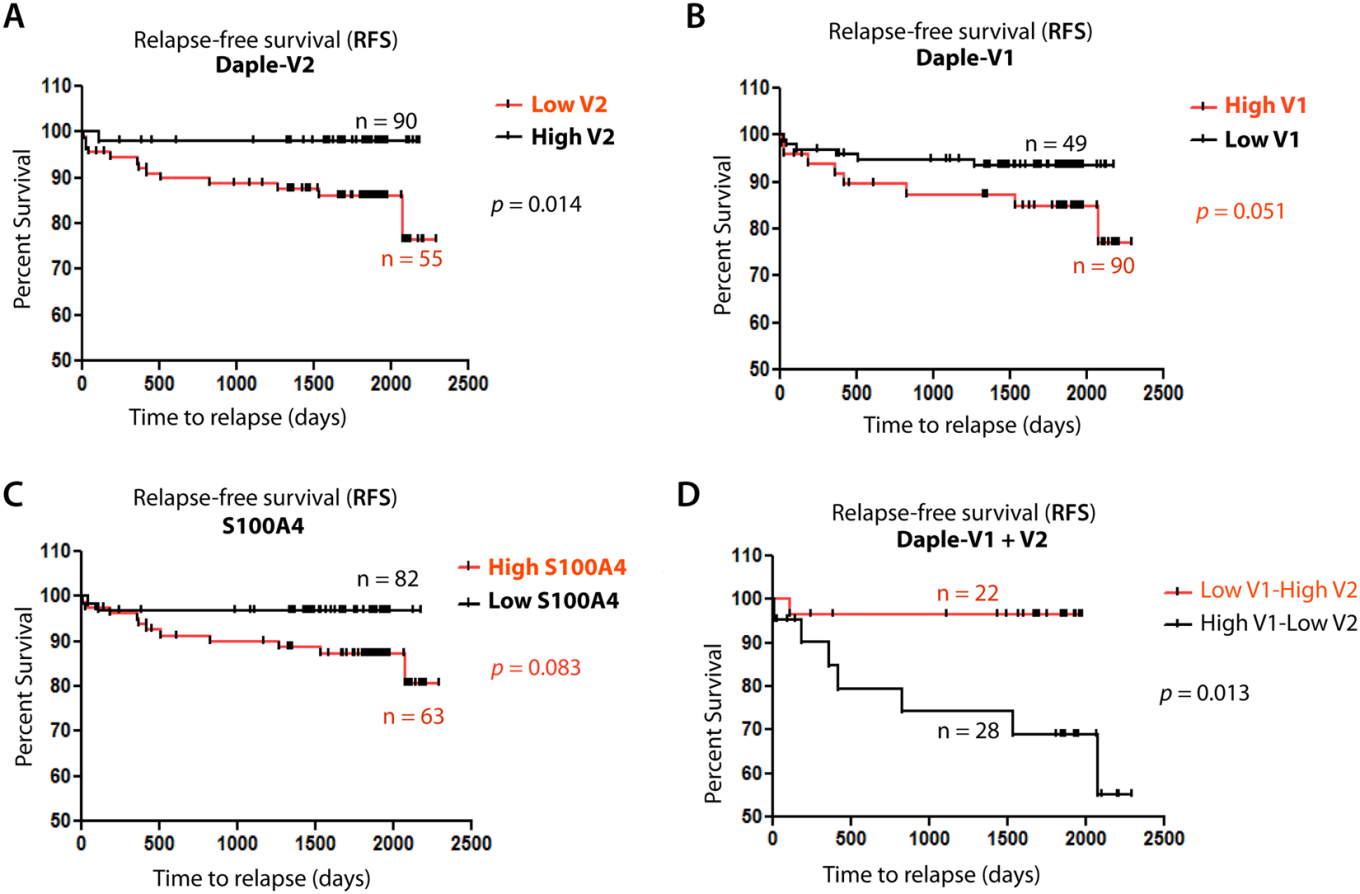
Association of relapse-free survival (RFS) with expression level of Daple isoforms V2 and V1 (**A**,**B**,**D**) and S100A4 (**C**). Kaplan-Meier plots for RFS (% survival; y-axis) of 145 patients with early stage melanoma was stratified based on levels of Daple-V2 alone (**A**) Daple-V1 alone (**B**) S100A4 alone (**C**) and Daple-V1 + V2 combined (**D**) and plotted against duration of follow-up (days to progression; x-axis). Although high Daple-V2, low Daple-V1 and low S100A4 carried better prognosis, statistical significance was reached only for Daple-V2. The positive prognostic impact of Daple-V2 and the negative prognostic impact of Daple-V1 were additive (**D**).

Because the frequency of metastatic progression was different for patients with high *vs*. low expression of transcripts for Daple-V1 or -V2 in the peripheral circulation at an early stage of disease (stage 0, I, II), we asked if levels of these transcripts can prognosticate relapse-free survival (RFS). Kaplan-Meier curves for high *vs*. low levels of expression of Daple-V1 and -V2 were plotted and compared between the two groups using the log rank test. In the case of Daple-V2, we found that RFS for patients with low expression was significantly reduced compared to patients with high expression (*p* = 0.014; Fig. 6A). While in the case of Daple-V1, we found that patients with high expression tend to have shorter RFS compared to patients with low expression (*p* = 0.051; Fig. 6B). A similar pattern was found in RFS analysis of S100A4; patients with high expression tend to have shorter RFS compared to patients with low expression (*p* = 0.083; Fig. 6C), but in both cases did not reach significance. These results indicate that Daple-V2, but not V1is a favorable prognostic indicator of RFS. Next we asked if the prognostic impact of Daple-V1 and Daple-V2 are additive. When we compared RFS between the group with low Daple-V1/high Daple-V2 and the group with high Daple-V1-low Daple-V2, we found that patients with low Daple-V1/high Daple-V2 have a much higher RFS than the other group (*p* = 0.013; Fig. 6D), indicating that Daple-V1 improves the prognostic value of Daple-V2.

### Stratification of patients by accounting for the expression levels of both Daple-V1 and Daple-V2 offers novel prognostic signatures

**Figure 7:**
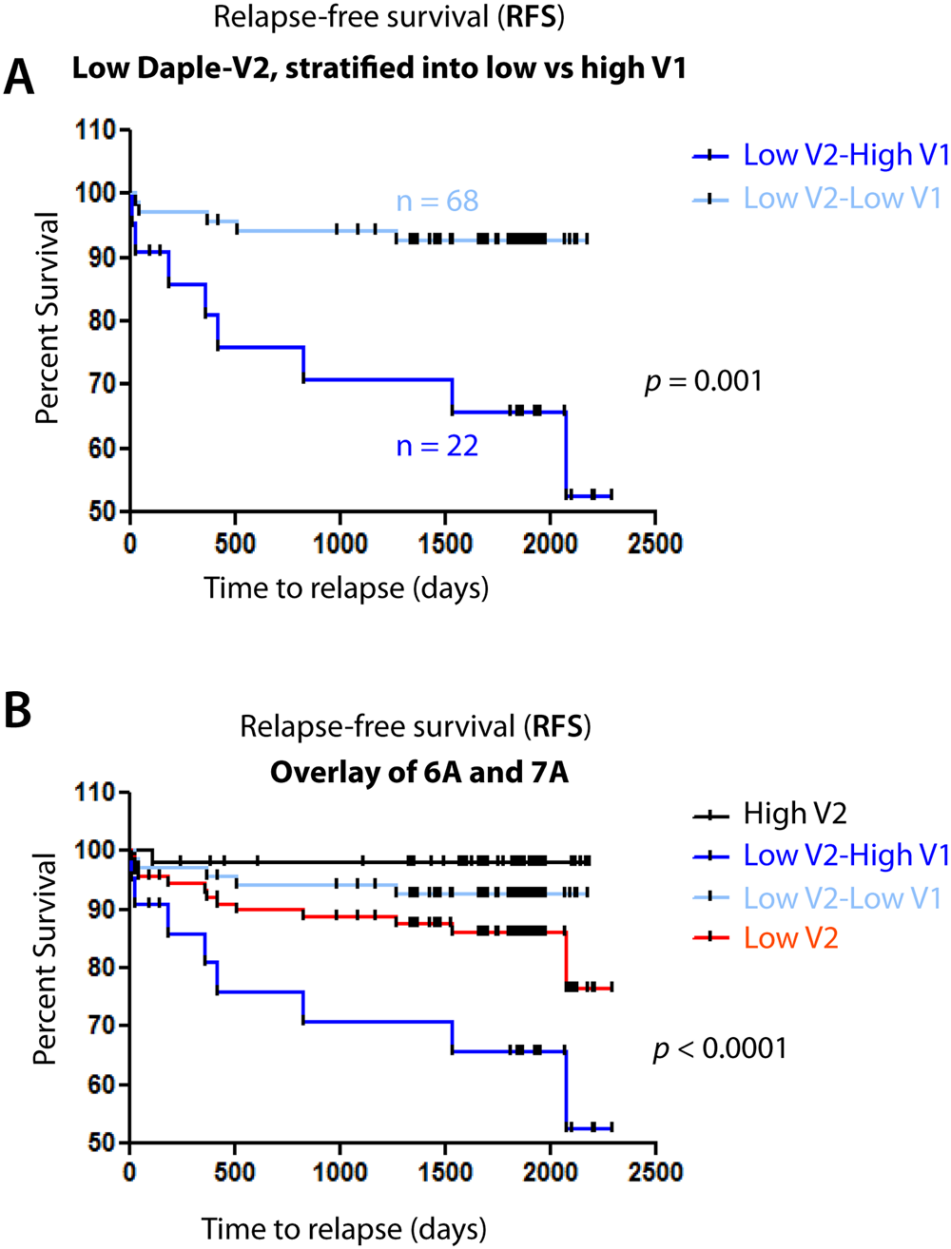
Stratification of patients with low Daple-V2 based on their levels of expression of Daple V1 further improves the prognostic power of Daple-V2. (**A**) Kaplan-Meier plots for RFS (% survival; y-axis) of 90 patients with early stage melanoma with low levels of Daple-V2 expression was stratified based on levels of expression of Daple-V1. (**B**) An overlay of Kaplan-Meier plots displayed in Fig. 6A [i.e., RFS among patients stratified based on Daple-V2 alone] and Fig. 7A.

To identify the most effective diagnostic algorithm that combines the prognostic impacts of both Daple isoforms, we explored if assessment of Daple-V1 among the patients with low Daple-V2 can improve the prognostic impact of Daple-V2. When we stratified patients with low Daple-V2 into two groups with high and low Daple-V1, we found that patients with high expression of Daple-V1 have shorter RFS than those with low expression of Daple-V1 (*p* = 0.001; Fig. 7A). RFS among patients with low Daple-V2-high Daple-V1 expression was significantly lower than those with high Daple-V2 expression (*p* < 0.0001; Fig. 7B).

We further evaluated the effect of Daple-V1 and -V2 on survival rates by univariate and multivariate analyses after adjustment for clinical covariates including age and tumor stage using Cox proportional hazard regression model (Cox analyses; Tables 2 and 3**)**. We found that Daple-V2 can be an independent prognostic factor; RFS of patients with low Daple-V2 expression was significantly lower than those with high Daple-V2 (hazard ratio [HR] 8.4006; *p* = 0.0403; 95% confidence interval [CI]1 1.0988 to 64.2249) in univariate analysis and hazard ratio [HR] 8.8907; p = 0.0366; 95% confidence interval [CI] 1.1462 to 68.9638 in multuvariate analysis). Univariate analyses also confirmed that Daple-V1 and Daple-V2 have additive prognostic value; RFS was significantly reduced when patients have high Daple-V1 and low Daple-V2 (HR 9.8416; *p* = 0.0325; confidence interval [CI] 1.2100 to 80.0448). However, multivariate analysis didn’t show the additive prognostic value of Daple-V1 and V2. The reason could be that the expression levels of Daple-V1 and V2 are not associated with age and the expression level of Daple-V1 is not associated with stages. Thus when taken together these unassociated variables for analysis, the power of a single variable is weakened. Although statistical significance was reached in each case, it is noteworthy that the 95% CIs are wide and further studies with larger cohort sizes are warranted. Regardless, both log-rank and Cox analyses are in agreement that Daple-V2 alone is a sufficient prognostic factor, and that risk stratification for tumor progression improves by the additive effect of Daple-V1 and Daple-V2. Risk was lowest when patients have low Daple-V1-high Daple-V2 signature, and higher when patients have a high Daple-V1-low Daple-V2 signature.

**Table 2:** Univariate analysis.

Univariates	*p-value*	Hazard Ratio [HR]	95% confidence interval [CI]
Low Daple-V2	0.0403	8.4006	1.0988 to 64.2249
High Daple-V1	0.1116	2.3987	0.8164 to 7.0472
Low Daple-V2 + High Daple-V1	0.0325	9.8416	1.2100 to 80.0448

**Table 3:** Multivariate analysis.

Covariate	*p-value*	Hazard Ratio [HR]	95% confidence interval [CI]
Low Daple-V2	0.0366	8.8907	1.1462 to 68.9638
Age	0.0773	0.9654	0.9283 to 1.0039
Stage	0.0027	4.3313	1.6647 to 11.2692
High Daple-V1	0.1563	2.1933	0.7404 to 6.4973
Age	0.1007	0.971	0.9376 to 1.0057
Stage	0.0028	4.0887	1.6221 to 10.3061
Low Daple-V2 + High Daple-V1	0.0468	8.4	1.0303 to 68.4825
Age	0.6672	0.99	0.9456 to 1.0364
Stage	0.0801	3.247	0.8685 to 12.1400

## Conclusions

The most important discovery we report here is a unique Daple/CCDC88C transcript signature, ‘high Daple-V1 and low Daple-V2’ in the peripheral blood of patients with early stage cutaneous melanoma that effectively stratifies patients at the highest risk for progression to metastatic disease. Daple-V1 triggers EMT via enhancement of non-canonical (β-catenin-independent) Wnt signals and suppresses canonical (β-catenin-dependent) Wnt signals^18^, whereas Daple-V2 serves primarily as a potent tumor suppressor^22^. Therefore, an increase in Daple-V1 at early stages in the peripheral blood (most likely from CMCs), suggests that non-canonical Wnt signals are likely enhanced and canonical Wnt signals are suppressed early during melanoma progression, a signature that has been previously described as a contributor to metastatic features of melanoma and poor survival^15–17,32^. A decrease in the tumor suppressive isoform, Daple-V2 at late stages suggests that canonical Wnt signals are upregulated and may be necessary for the growth of metastatic lesions at distant sites. Overall, an increase in Daple-V1 and a decrease in Daple-V2 expression correlated with disease progression and emerged from this work as the most effective signature in predicting the risk for metastatic progression. Although, this study did not shed light into if and how tumor microenvironment may dictate the relative levels of these two transcripts during melanoma progression, what is intriguing that this signature also appears to have an overall permissive signaling program in colon cancer cells, aiding both tumor cell proliferation (due to low Daple-V2) and EMT (due to high Daple-V1)^22^.

**Table 4:** Cut-off values used for various transcripts.

Transcript name	Cut-off valuefor high/low level
Daple-V2	0.7393585
Daple-V1	1.267289
S100A4	1.7226087

It is noteworthy that although lower Daple-V2 transcripts in the peripheral circulation were seen predominantly in patients with late stage disease, lower Daple-V2 transcripts in the early stage of the disease emerged as the strongest predictive profile in our study. This came as an unusual surprise to us because typically biomarkers representative of CTCs are elevated in circulation over background, not decreased. These findings raise the possibility that circulating melanocytes may be present in the peripheral circulation of healthy controls, and hence, Daple-V2 transcript is detected in the circulation of normal healthy individuals. However, during metastatic progression of melanoma, different populations of CMCs that have low Daple-V2 may be disseminated. In fact, this possibility is supported by prior studies that reported that melanocyte precursor cells – cells which express TYR and MITF transcripts, but not MLANA (or MIF) - may indeed circulate in healthy adults with benign nevus, are detected in human cord blood^33–35^, and during melanocytic differentiation into vein-like structures from pluripotent stem cells^36^. In patients with melanoma, a shift is noted in the landscape of the melanoma transcriptome, i.e., MLANA and MIF transcripts are elevated (but not TYR or MITF)^34^, leading some to speculate that the circulation of normal melanocytic precursors is inhibited in patients with melanomas, perhaps due to factors, e.g., cytokines released into the systemic circulation^34^. Based on these studies, and our own findings, we conclude that the entry of normal melanocytes and/or its precursors that have high Daple-V2 is inhibited in patients with melanoma, and instead, melanoma cells with lower Daple-V2 begin to enter the circulation. Because the burden of metastatic CMCs in the circulation increases with increasing clinical stage of disease^5^, the prognostic value of decreased Daple-V2 transcript that we observe in the early stage suggests that it may be a sensitive method to detect the changing landscape of melanocyte transcripts and serve as a surrogate marker for the overall load of metastatic CMCs.

Overall, we conclude that the abundance of Daple transcripts (V1 and V2) in the peripheral blood of patients with melanoma may serve as a clinically useful early prognostic test for detecting CMCs in the circulation. Because malignant melanoma is a disease that can be characterized by prolonged periods of tumor dormancy, which necessitates prolonged periods of follow-up, monitoring Daple transcripts in the peripheral circulation may serve as a relatively simple minimally-invasive test, either administered stand-alone, or in combination with other panels of melanoma markers. Further studies in larger cohorts are necessary before one can rigorously assess and realize the clinical usefulness of Daple in melanoma surveillance during follow-up, to detect tumor progression, to monitor response to therapy, and therefore to guide treatment plans.

## Materials and Methods

### Patient cohort

All samples used in this study were previously analyzed as part of a prior study^7^, the details of which have been well documented. All research was performed in accordance with relevant guidelines/regulations imposed by the IRB. Written informed consent was obtained from 205 patients with primary cutaneous melanoma and 142 healthy control participants for this study^7^. Of the 205, 145 had American Joint Committee on Cancers (AJCC) clinical stages 0, I or II (i.e., early stage) and 60 patients had AJCC stages III or IV (i.e., late stage). The majority of these patients had no adjuvant therapy (19 patients, 51%). The remaining patients received either a combination of post-operative radiation and Dacarbazine (DTIC) treatment (4 patients, 11%), only post-operative radiation (10 patients, 27%), a combination of post-operative radiation and a melanoma vaccine (1 patient, 2.75%), a trial BRAF inhibitor (1 patient, 2.75%), Interferon alpha 2b and limb infusion (1 patient, 2.75%) or a combination of DTIC, PI-88 and ticilimumab (1 patient, 2.75%). The Human Research Ethics Committees of Edith Cowan University (No. 2932) and Sir Charles Gairdner Hospital (No. 2007-123) approved the study. Patients were recruited from the Medical Oncology Department of Sir Charles Gairdner Hospital and the Perth Melanoma Clinic at Hollywood Hospital (Perth, Western Australia), while the aged-matched, healthy group was recruited from the general population. Detailed patient cohort information is listed in Table 1.

### Sample collection

As reported previously^7^, 2.5 ml of whole blood was collected into a PAXgene Blood RNA Tube (PreAnalytiX, Hombrechtikon, Switzerland) containing RNA stabilizers. Total RNA was isolated using the PAXgene Blood RNA Kit (Qiagen, Hilden, Germany) and then reverse transcription was performed by Omniscript Reverse Transcriptase (Qiagen). Clinical data and post-blood collection follow-up was collected for all patients over 3 years and 9 months.

As for the timing of sample collection, for the Stage 0, Stage I and Stage II patients underwent venipuncture *after* their curative intent surgery, i.e., after their primary lesion was removed or following a wider excision of their primary lesion. Briefly, 43.6% of Stage 0 patients (N = 17) were sampled after a wider excision and 56.4% (N = 22) after their primary lesion was removed. Patients were sampled on average 33.5 months *after* the removal of their primary lesion (range 0–168 months) or on average 18.76 months after their wider excision (range 0–56 months). Among Stage I patients, 35% (N = 28) had blood collected after a wider excision and 65% (N = 52) after their primary lesion was removed. Patients were sampled on average 47 months after the removal of their primary lesion (range 0–310 months) or on average 44.86 months after their wider excision (range 0–225 months). Among Stage II patients, venipuncture occurred after a wider excision for 52.5% (N = 21) of Stage II patients, with the remaining 47.5% (N = 19) of Stage II patients sampled after their primary was removed. Patients were sampled on average 84 months after the removal of their primary lesion (range 0–1239 months) or an average of 27.76 months after their wider excision (range 0–160 months).

As for Stage III patients, they were sampled *after* the removal of their lymphatic lesions; however, some patients went on to develop metastatic lesions in other organs (stage IV) or further lymphatic lesions. Patients were sampled on average 12.96 months after the removal of their lymphatic lesion/s (range 0–52 months). Finally, for Stage IV patients, 43.2% (N = 16) of them had already undergone surgery for metastatic disease when their blood was collected. Patients were sampled on an average of 9.9 months after their surgery (range 0–64 months). The remaining 56.8% (N = 21) of Stage IV patients had active disease at the time of blood collection with unremoved metastases present.

### Melanoma cell lines, RNA isolation, standard curve and quantitative PCR (qPCR)

Human melanoma cell line A375 (obtained from ATCC) was grown in Dubecco’s modified Eagle’s medium (DMEM; Gibco) media containing 10% fetal bovine serum (Hyclone) in T75 cm^2^ flask (Corning) until cells were ~70% confluent. Cells were then collected and total RNA was isolated using an RNeasy kit (QIAGEN) as per the manufacturers’ protocol. First-strand cDNA was synthesized using Superscript II reverse transcriptase (Invitrogen), followed by ribonuclease H treatment (Invitrogen) prior to performing quantitative real-time PCR. A standard curve, to quantify mRNA copy number, was constructed using larger PCR products (~500 bp) that included the target sequence used in qPCR. Reactions omitting reverse transcriptase were performed in each experiment as negative controls. Reactions were then run on a real-time PCR system (ABI StepOnePlus; Applied Biosystems). Gene expression of Daple-V1, Daple-V2 and S100A4 were detected with Taqman assay (Invitrogen), and relative gene expression was determined by normalizing to GAPDH using Relative standard curve method. Primer and probe sequences are as listed in Supplementary Table 1 (Table S1).

### Analysis of melanoma-associated markers

These analyses were carried out as described previously^7^. Briefly, total RNA (250 ng) was converted to cDNA (Omniscript Reverse Transcriptase, Qiagen), and included as a no template control. qRT-PCR assay assessed the number of mRNA transcripts (level of expression) for three genes that have been previously shown to be associated with melanoma cells, and in some cases, indicative of stemness, i.e., MLANA, ABCB5 and MCAM. GAPDH was assessed as an internal loading control because its level is not upregulated in melanoma tissues or cultured cells relative to normal samples^37^. These assays were carried out using SYBR GreenER qPCR SuperMix (Invitrogen) and 200 nmol L^−1^ of primer (Table S1) and an iCycler iQ5 Real-Time Thermocycler (Bio-Rad, Hercules, CA, U.S.A.) as per the manufacturer’s instructions. To prevent contamination, all PCR reactions contained uracil-N-glycosylase to prevent reamplification of carryover PCR products, and different steps were performed in separate ultraviolet-treatable areas. Melting point determination and gel electrophoresis confirmed the expected size and identity of PCR products. Every assay included a standard curve, negative controls (no template and reverse transcription control), positive control (A2058 cell line) and cDNA from a single healthy control.

### Statistical analyses

Statistical evaluation was performed using the GraphPad prism v5 software. Statistical significance of differences in marker expression among the patients with early *vs*. late stages of melanoma and normal healthy controls was analyzed using unpaired t-test. Optimal cut-off values for gene expression levels were derived using the web-based tool-Cutoff Finder (R Software v2.15.0^38^) by maximally selected log-rank statistics. We chose the method–‘*Significance of correlation with survival variable*’ for cutoff optimization over ‘ROC curve analysis’ from Cutoff Finder because this method fits Cox proportional hazard models to dichotomized variables and survival variables which is executed using the functions *coxph* and *survfit* from the survival package. The optimal cutoff is defined as the point with the most significant (log-rank test) split. The relationship between high *vs*. low levels of gene expression and incidence of metastatic progression was investigated by Fisher’s exact test. The association between melanoma stage and expression level of Daple-V1/V2 and S100A4 was carried out by Chi-square test for trend. Cox proportional hazard regression analysis was used to generate hazard ratio and confidence interval of transcrips. To assess the relationship between gene expression and patient’s age, clinical stage of disease, Clark level, Breslow thickness and ulceration, we usedparametric Pearson’s correlation and nonparametric Spearman’s correlation analyses. To estimate the correlation among the expression levels of various genes tested here, we used Correlation Matrix. To analyze the effect of high *vs*. low levels of gene expression and time-dependent survival probabilities we used Kaplan-Meier (KM)analyses. All statistical tests were performed two-sided, and *p*- values less than 0.05 were considered as statistically significant.

## Electronic supplementary material

supplementary file

## Data Availability

All data generated or analyzed during this study are included in this published article (and its Supplementary Information Files). The raw datasets generated during and/or analyzed during the current study are available from the corresponding author on reasonable request. The TCGA datasets analyzed during the current study are available in the cBioPortal repository, [http://www.cbioportal.org/].
