# Supplementary material for: Prognostic Relevance of CCDC88C (Daple) Transcripts in the Peripheral Blood of Patients with Cutaneous Melanoma
Source: Sci Rep. 2018 Dec 21;8:18036. doi: 10.1038/s41598-018-36173-x (PMC6303298; doi:10.1038/s41598-018-36173-x)
Supplement: Supplementary file 1 — supplementary file [file 41598_2018_36173_MOESM1_ESM.pdf]

**SUPPLEMENTARY ONLINE MATERIALS**

# Prognostic Relevance of CCDC88C (Daple) Transcripts in the Peripheral Blood of Patients with Cutaneous Melanoma

Ying Dunkel[1], Anna L. Reid[2], Jason Ear[1], Nicolas Aznar[1, 3], Michael Millward[4,5], Elin Gray[2], Robert Pearce[2], Melanie Ziman[2, 6]¶ and Pradipta Ghosh[1,7, 8]¶ *

[1]Department of Medicine, University of California, San Diego, La Jolla, California, USA.
[2]School of Medical Sciences, Edith Cowan University, Perth, WA, Australia
[3] Centre de Recherche enCancérologie de Lyon (CRCL), Lyon, France.
[4]School of Medicine, University of Western Australia, Crawley, Australia
[5]Department of Medical Oncology, Sir Charles Gairdner Hospital, Nedlands, Australia
[6]School of Biomedical Science, University of Western Australia, Crawley, Australia
[7]Department of Cellular and Molecular Medicine, University of California, San Diego, La Jolla, California, USA.
[8]Rebecca and John Moores Cancer Center, University of California, San Diego, La Jolla, California, USA.

**Supplementary Table 1 [Table S1]**

| Target gene | Primer and Probe sequence **(TAQMAN ASSAY)** |
| --- | --- |
| Daple-V1 | Fwd: 5'-CGGGACCTCACCAAGCAA-3' |
| | Rev: 5'-CTGCTGAGCTGCTGGCTCTT-3' |
| | Probe: 5'-CAACTCTGAGGGAGGACCTGGTGCTC-3' |
| Daple-V2 | Fwd: 5'-GGAGCCTCAGGATATACGTGCA-3' |
| | Rev: 5'-TCAAGGCTGCCTCTGTGTGG -3' |
| | Probe: 5'-CAGGATGTCCGTACTAAGCCCTGGGGATC-3' |
| S100A4 | Fwd: 5'-CAAGCTCAACAAGTCAGAACTAAAGGA-3' |
| | Rev: 5'-TGTCCAAGTTGCTCATCAGCTTC-3' |
| | Probe: 5'- CCAGCTTCTTGGGGAAAAGGACAGATGAA-3' |
| GAPDH | Fwd: 5'- CCAATGTCACCGTTGTCCAGTT-3' |
| | Rev: 5'- CTCTACTTCAGCCTTAGGCTCAGGT-3' |
| | Probe: 5'-CCGTGCCCGGTGACCAGAGC-3' |

| Target gene | Primer sequence **(SYBR-GREEN ASSAY)** |
| --- | --- |
| MLANA | Fwd: 5'-GAGAAAAACTGTGAACCTGTGGT -3' |
| | Rev: 5'-GACTGTTCTGCAGAGAGTTTCTCAT-3' |
| ABCB5 | Fwd: 5'-CCCAAAATTTTATTGTTGGATGA-3' |
| | Rev: 5'-AAGGGCATGCTGAACCAC-3' |
| MCAM | Fwd: 5'-GGGTACCCCATTCCTCAAGT-3' |
| | Rev: 5'-CTGGGACGACTGAATGTGG-3' |
| GAPDH | Fwd: 5'-GGGTGTGAACCATGAGAAGT-3' |
| | Rev: 5'-GACTGTGGTCATGAGTCCT-3' |

**Supplementary Table 2 [Table S2]**

| | | DAPLE-V1 | DAPLE-V2 | S100A4 |
|---|---|---|---|---|
| DAPLE-V1 | Spearman r | 1.0000 | 0.5466 | 0.8014 |
| | *p*-value | n/a | <0.0001 | <0.0001 |
| DAPLE-V2 | Spearman r | 0.5466 | 1.0000 | 0.3440 |
| | *p*-value | <0.0001 | n/a | <0.0001 |
| S100A4 | Spearman r | 0.8014 | 0.3440 | 1.0000 |
| | *p*-value | <0.0001 | <0.0001 | n/a |

**Supplementary Table 3 [Table S3]**

| | GENES | DAPLE-V1 | DAPLE-V2 | S100A4 |
|---|---|---|---|---|
| AGE | Spearman r | 0.04293 | 0.03299 | 0.1831 |
| | *p*-value | 0.5411 | 0.6387 | **0.0086** |
| STAGE | Spearman r | -0.05526 | -0.1624 | -0.1327 |
| | *p*-value | 0.4313 | **0.02** | 0.0578 |
| CLARK'S LEVEL | Spearman r | -0.0414 | -0.1039 | -0.09071 |
| | *p*-value | 0.579 | 0.1628 | 0.2233 |
| BRESLOW THICKNESS | Pearson r | 0.121 | 0.01823 | -0.00617 |
| | *p*-value | 0.1515 | 0.8295 | 0.9419 |
| ULCERATION | Spearman r | -0.02815 | 0.0732 | -0.08334 |
| | *p*-value | 0.7006 | 0.3168 | 0.2542 |

**Supplementary Table 4 [Table S4]**

**A**

| MARKERS STATUS (MLNA, ABCB5 OR MCAM) | EARLY STAGE PATIENTS WITH LOW V2(N) | EARLY STAGE PATIENTS WITH HIGH V2 (N) | TOTAL (N) |
|---|---|---|---|
| **MARKERS (+) (%)** | 48 (64%) | 29 (41%) | 77 |
| **MARKERS (-) (%)** | 27 (36%) | 41 (59%) | 68 |
| **TOTAL (N)** | 75 | 70 | 145 |

**B**

| ABCB5 STATUS | PATIENTS WITH LOW V2 (N) (ALL STAGES) | PATIENTS WITH HIGH V2 (N) (ALL STAGES) | TOTAL (N) |
|---|---|---|---|
| **ABCB5 (+) (%)** | 37 (27%) | 9 (13%) | 46 |
| **ABCB5 (-) (%)** | 99 (73%) | 60 (87%) | 159 |
| **TOTAL (N)** | 136 | 69 | 205 |

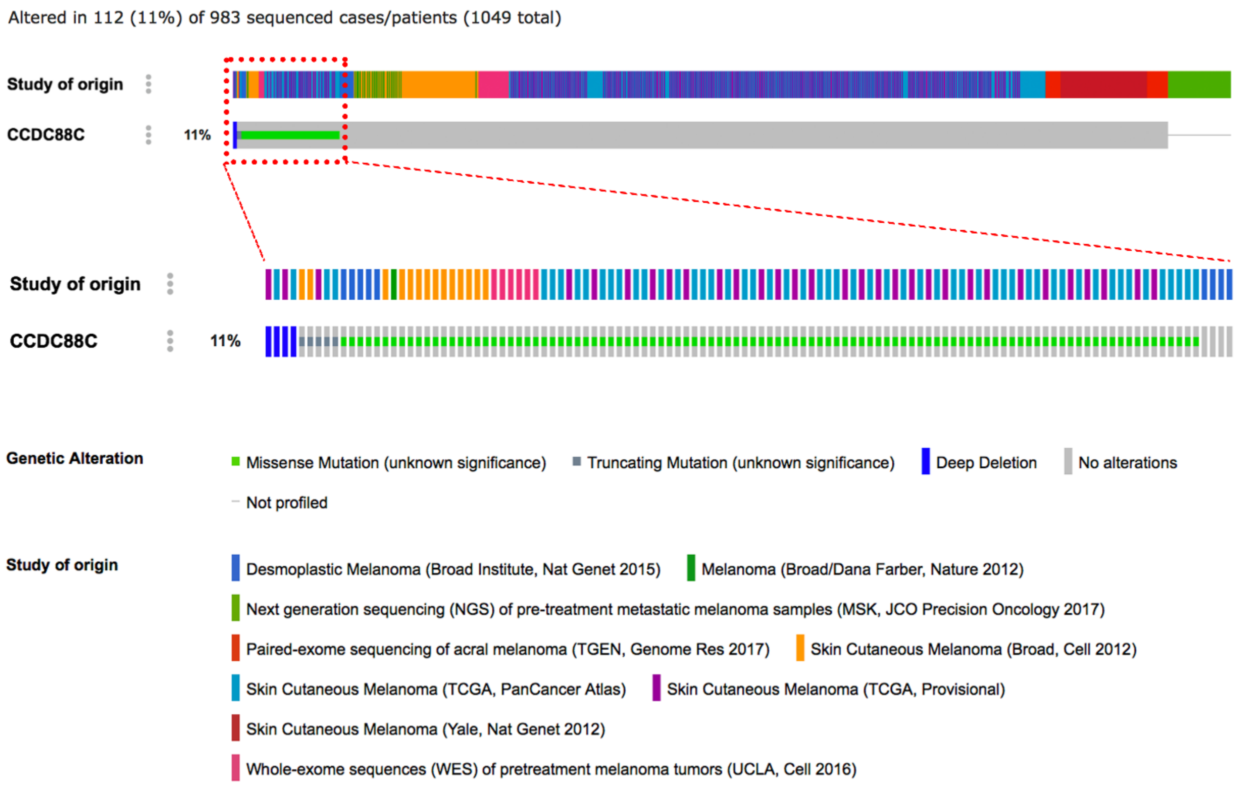


## Supplemental Figure 1

**Summary of CCDC88C/ Daple gene alteration across multiple melanoma datasets.**
Approximately 11% of patients carry a mutation in the gene. Missense mutations, truncating mutations, and homozygous deletions (deep deletions) have been identified in patients, with missense mutations accounting for most of the mutations.

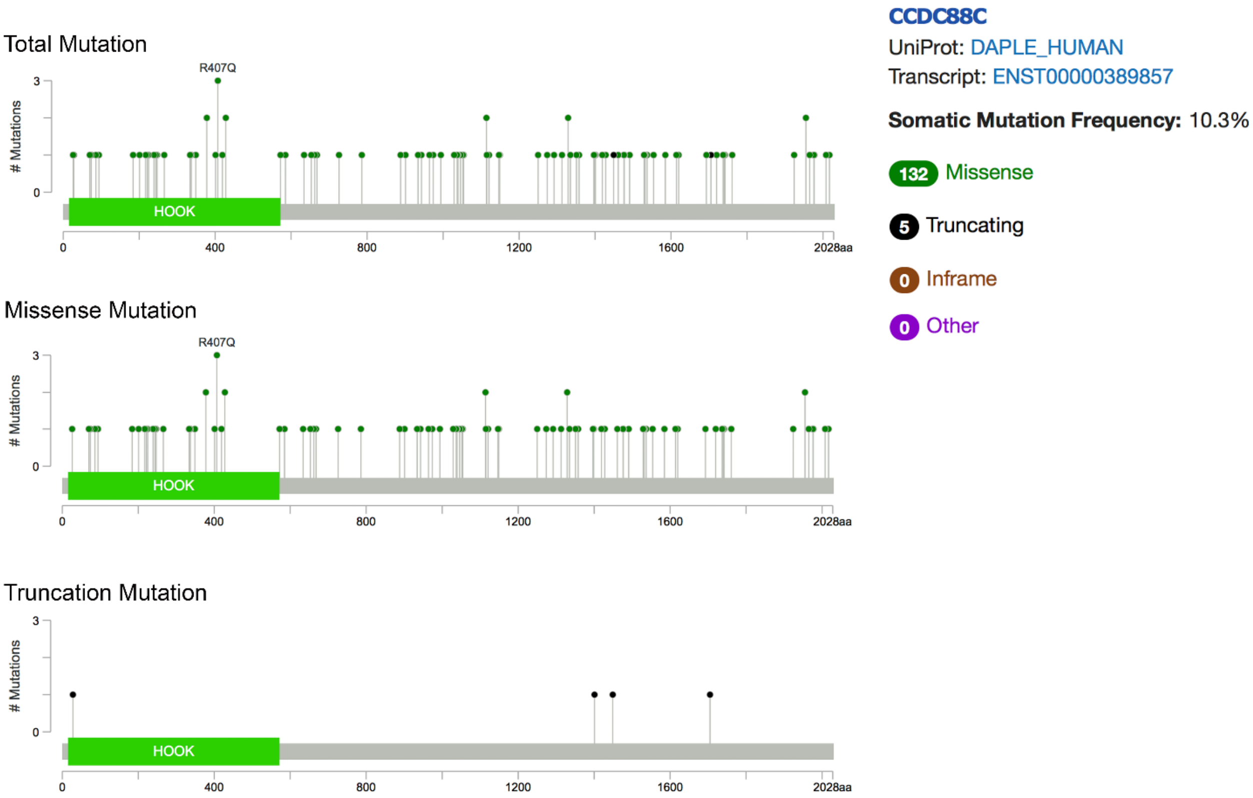


**Supplemental Figure 2**

**Daple is frequently mutated in melanomas.** Mutation diagram summarizing the number of mutations, mutation rate, and position of the mutation along the Daple protein in melanomas. The total landscape of mutation is illustrated in the top plot. Middle plot illustrates exclusively the missense mutations, whereas the bottom plot illustrates exclusively the truncating mutations.

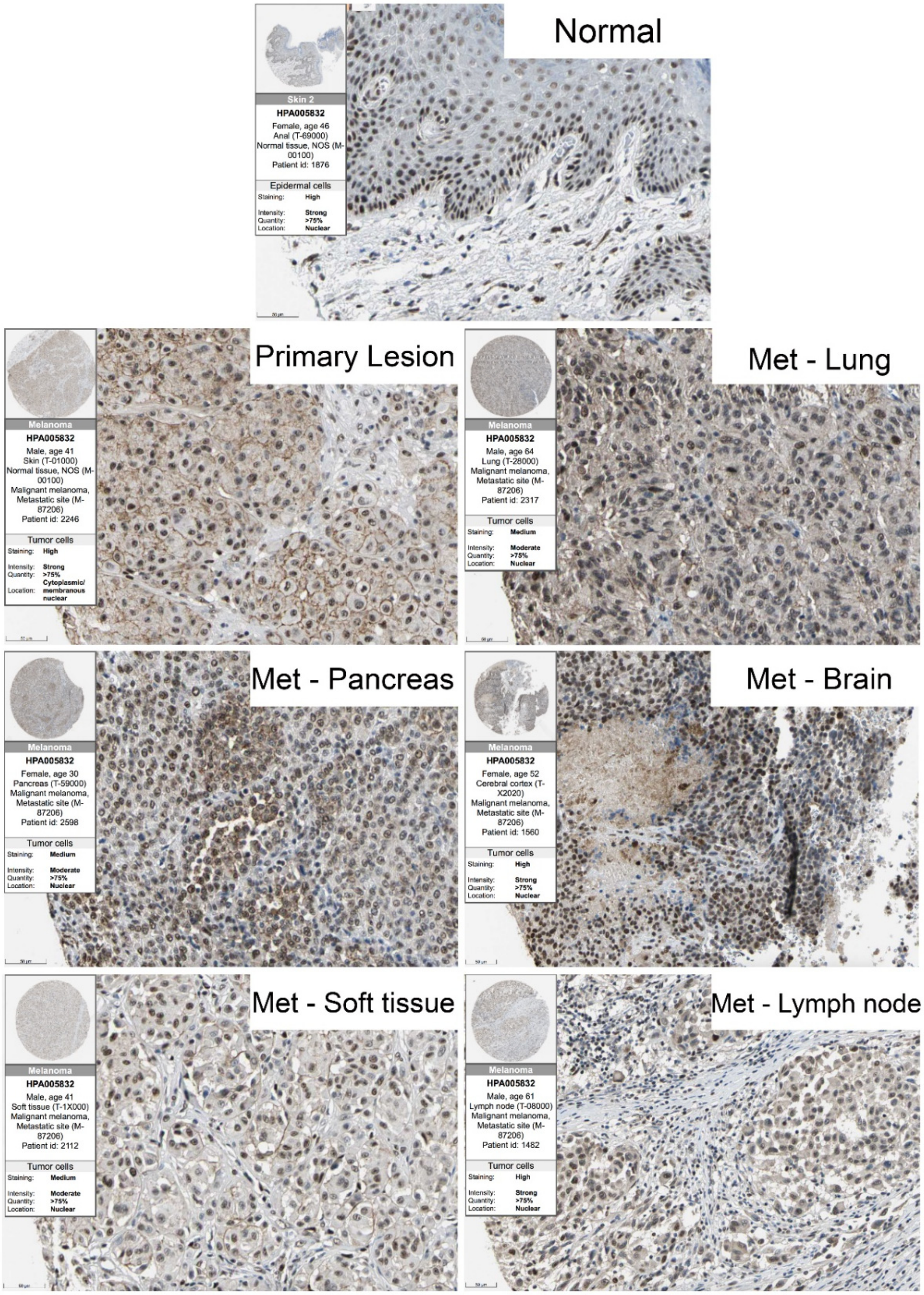


**Supplemental Figure 3**

**Daple is highly expressed in cutaneous melanomas.** Expression of Daple, as determined by immunohistochemistry (IHC) on formaldehyde fixed paraffin embedded (FFPE) normal skin and primary and metastatic melanoma tissues as reported in The Human Protein Atlas (using antibody HPA005832). Of a total of 11 tumors, a 100% of the analyzed samples showed high (6) or very high (5) levels of expression. *Met*, Metastatic Site.

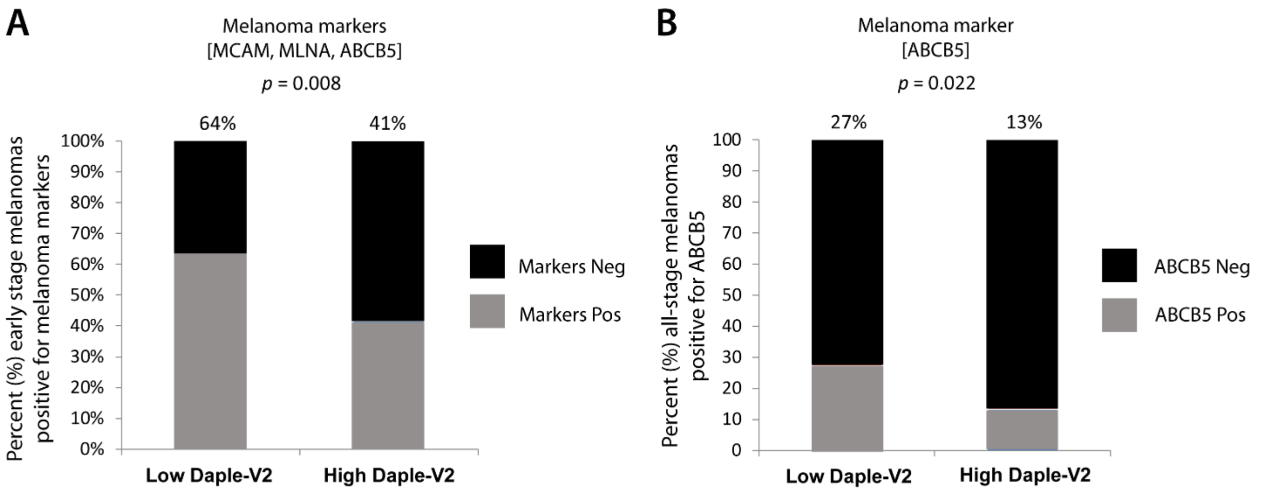


**Supplemental Figure 4**

**Correlation between Daple-V2 expression and melanoma-associated markers.** (A) Patients with early stage melanomas (n = 145) with known expression status (positive or negative) of melanoma associate markers (MCAM, MLNA and ABCB5) were analyzed in subgroups of patients with high (n = 55) or low (n = 90) expression levels for Daple-V2. Tumors were classified as "Markers Pos" if any one of the three melanoma-associated markers were detectable, whereas tumors were classified as "Markers Neg" if none of the three markers were detectable [see **Table S4-source data**]. Bar graphs display the incidence of marker positivity as percent (%; Y axis) of patients with low *vs*. high Daple-V2. (**B**) Patients with all stages of melanoma (n = 205) with known expression status (positive or negative) of the melanoma stem cell marker, ABCB5 alone was analyzed in subgroup of patients with low *vs*. high Daple-V2 as in **A**.
